# Conditional Deletion of *Fgfr1* in the Proximal and Distal Tubule Identifies Distinct Roles in Phosphate and Calcium Transport

**DOI:** 10.1371/journal.pone.0147845

**Published:** 2016-02-03

**Authors:** Xiaobin Han, Jiancheng Yang, Linqiang Li, Jinsong Huang, Gwendalyn King, L. Darryl Quarles

**Affiliations:** 1 Department of Medicine, University of Tennessee Health Science Center, Memphis, Tennessee, United States of America; 2 University of Alabama in Birmingham, Birmingham, Alabama, United States of America; Aarhus University, DENMARK

## Abstract

A postnatal role of fibroblast growth factor receptor-1 (FGFR1) in the kidney is suggested by its binding to α-Klotho to form an obligate receptor for the hormone fibroblast growth factor-23 (FGF-23). FGFR1 is expressed in both the proximal and distal renal tubular segments, but its tubular specific functions are unclear. In this study, we crossed *Fgfr1*^flox/flox^ mice with either gamma-glutamyltransferase-Cre (*γGT-Cre*) or kidney specific-Cre (*Ksp-Cre*) mice to selectively create proximal tubule (PT) and distal tubule (DT) *Fgfr1* conditional knockout mice (designated *Fgfr1*^*PT-cKO*^ *and Fgfr1*^*DT-cKO*^, respectively). *Fgfr1*^*PT-cKO*^ mice exhibited an increase in sodium-dependent phosphate co-transporter expression, hyperphosphatemia, and refractoriness to the phosphaturic actions of FGF-23, consistent with a direct role of FGFR1 in mediating the proximal tubular phosphate responses to FGF-23. In contrast, *Fgfr1*^*DT-cKO*^ mice unexpectedly developed hypercalciuria, secondary elevations of parathyroid hormone (PTH), hypophosphatemia and enhanced urinary phosphate excretion. *Fgfr1*^*PT-cKO*^ mice also developed a curly tail/spina bifida-like skeletal phenotype, whereas *Fgfr1*^*DT-cKO*^ mice developed renal tubular micro-calcifications and reductions in cortical bone thickness. Thus, FGFR1 has dual functions to directly regulate proximal and distal tubule phosphate and calcium reabsorption, indicating a physiological role of FGFR1 signaling in both phosphate and calcium homeostasis.

## Introduction

There are 22 mammalian fibroblastic growth factors (FGFs) subgrouped into 7 subfamilies and four alternative spliced FGF receptor genes (FGFR1-FGFR4) that encode seven membrane-associated tyrosine kinase isoforms (FGFRs 1 b, 1c, 2b, 2c, 3b, 3c and 4). The majority of FGFs are retained in tissues and act as paracrine/autocrine activators of FGFRs in the presence of heparin sulfate proteoglycans. The more recently evolved subfamily of hormonal FGFs, include FGF-19, FGF-21 and FGF-23, have N-terminal FGF-homology domains linked to novel C-termini [1–3] that allow entry into the circulating and binding to a receptor complex formed by FGFRs and Klotho (KL), a type I membrane ß-glycosidase-like protein.

Hormonal FGFs have defined novel endocrine networks and brought new attention to the postnatal functions of FGFR signaling. One of these is the bone-kidney endocrine network, where FGF-23 produced by osteoblasts and osteocytes in bone regulates proximal (PT) and distal tubular (DT) functions in the adult kidney [1, 4–8]. In the PT, FGF-23 inhibits sodium-dependent phosphate co-transporter activity, leading to phosphaturia; and inhibits Cyp27b1-mediated 1-α-hydroxylation of 25(OH)D and stimulates Cyp24a1-mediated 24-hydroxylation of 1,25(OH)_2_D, leading to reductions in circulating 1,25(OH)_2_D [9]. More recent studies indicate that FGF-23 also has distal tubular functions that include stimulation of renal sodium and calcium retention [10–12].

There a several knowledge gaps in FGF-23 regulation of renal tubular functions. For example, specific FGFRs that mediate the effects of FGF-23 in the kidney, the necessity for α-KL co-expression, and the precise tubular functions of FGFR activation by FGF-23 remain unclear. FGFR1c, 3c or 4, but not FGFR2, can form complexes with α-KL to constitute a functional FGF-23 receptor [13–16]. Immunohistochemistry analysis of the kidney originally identified FGFR3 in the proximal tubule [17, 18], FGFR-1, - 3 and -4 in the distal tubules, and FGFR2 in the distal straight tubules [18]; however, recent studies using more sensitive RT-PCR found that FGFR-1 and -4 transcripts are also expressed in the proximal tubule [9, 19]. Mouse genetic approaches have so far failed to resolve the question of tubular specific functions of FGFRs [9, 20, 21]. Whereas *Fgfr3*^-/-^ or *Fgfr4*^-/-^ mice have no kidney phenotype, compound *Fgfr3*^*-/-*^/*Fgfr4*^*-/-*^ mice have increased serum 1,25(OH)_2_D levels, are completely resistant to FGF-23-mediated suppression of 1,25(OH)_2_D, and partially resistant to its phosphaturic actions [18, 20, 21]. *Fgfr1*^*-/-*^ mice are embryonic lethal, but conditional deletion of FGFR1 in both the PT and DT using *Pax3-Cre* resulted in partial inhibition of phosphaturic responses to rFGF-23 associated with an increase in Npt2c expression in the kidney, but no effect on calcium or vitamin D metabolism [9, 22]. Concomitant loss of FGFR4 and FGFR1 in *Pax3-Cre/Fgfr1*^*cKO*^ mice were required to disturb 1,25(OH)_2_D metabolism. While these studies show FGFRs have both overlapping and distinct functions in the kidney, they fail to define the tubular specific functions of individual receptors.

Uncertainty about the tubular expression of α-KL, the FGFR co-receptor, which imparts both function and cell selectivity to FGF-23 [13], further confounds the analysis of FGFR function in the kidney. Indeed, α-KL expression is mainly expressed in the distal tubule, but the principal function of FGF-23 is in the proximal tubule, a disparity leading to the conjecture of a distal-to-proximal paracrine feedback mechanism responsible for the proximal tubular effects of FGF-23 [20]. Subsequently, *α-Kl* transcripts were detected in the dissected PT segments, suggesting that FGF-23 directly activates FGFR/α-KL complexes in the proximal tubule [23], but whether FGF-23 directly activates FGFRs in the proximal tubule remains controversial. Indeed, signally responses after rFGF-23 administration are limited to the distal tubule [24]. Moreover, conditional deletion of α-KL from the DT using *Ksp-Cre* (*α-Kl*^*DT-cKO*^) results in hyperphosphatemia and increased expression of vitamin D regulatory enzymes in association with elevated FGF-23 levels, indicating that loss of α-KL in the distal tubule can affect proximal tubule functions [25].

Studies of *Fgf-*23^-/-^ and *α-Kl*^*-/-*^ mice also implicate FGF-23 in the regulation of distal tubular calcium transport, but the mechanism is controversial and the specific FGFRs mediating FGF-23 effects on distal tubule calcium have not been defined. FGF-23 activation of FGFR/α-KL co-receptors in the distal tubule is purported to stimulate TRPV5 trafficking and membrane insertion leading to increases calcium absorption [12]. On the other hand, FGF-23 suppresses the expression of α-KL, which stabilizes the membrane expression of TRPV5 through its glucuronidase/sialidase activity [26]. FGF-23-mediated reductions in α-KL and TRPV5 insertion should also inhibit distal tubular calcium reabsorption. Elucidating the mechanisms of FGF-23 effects on the distal tubule is confounded by limitations of *Fgf-23*^-/-^ and *Kl*^-/-^ mouse models, which have systemic alterations in 1,25(OH)_2_D and PTH that preclude distinguishing between FGF-23 dependent and independent effects on distal tubule calcium transport.

In the current study, we tested the selective function of FGFR1 in the proximal and distal tubule and its role in mediating the effects of FGF-23 on proximal and distal tubular functions in mice with conditionally deletion of *Fgfr1* from the either proximal or distal nephron segments. We confirm an important direct role of FGFR1 signaling in the regulation of phosphate transport in the proximal tubule. We also found an important role of FGFR1 in regulating distal tubule calcium transport. This suggests a new schema for FGF-23 physiological functions to coordinately regulate proximal and distal functions to maintain phosphate and calcium homeostasis.

## Methods and Materials

### Animals breeding and genotyping

All animal research was conducted according to guidelines provided by the National Institutes of Health and the Institute of Laboratory Animal Resources, National Research Council. The University of Tennessee Health Science Center's Animal Care and Use Committee approved all animal studies (Protocol number: 12–168.0). *CMV-*Cre mice were originally purchased from the Jackson Laboratory (Bar Harbor, ME, USA) and maintained in C57BL/6J background. The floxed *Fgfr1* mice (*Fgfr1* ^*flox/flox*^) were obtained from Dr. Chuxia Deng at National Institute of Diabetes and Digestive and Kidney Diseases and maintained in C57BL/6J background for at least six generations. *γGT-Cre* [27] and *Ksp-Cre* [28] mice were used to delete the floxed *Fgfr1* in kidney as described previously and maintained in C57BL/6J background for at least five generations. All mice were maintained on a standard diet (7912, Harlan Teklad, Madison, WI, USA). First, we crossed *Fgfr1*^*flox/+*^ to *CMV*-Cre to obtain a germline-specific deletion of *Fgfr1* (*Fgfr1*^null/+^). The *Fgfr1*^null/+^ mice were crossed to *γGT-Cre* mice or *Ksp-*Cre mice to obtain heterozygous *γGT-Cre;Fgfr1*^*null/+*^ or *Ksp-*Cre;*Fgfr1*^null/+^ mice, respectively. Then, *Fgfr1*^flox/flox^ females were crossed to *γGT-Cre;Fgfr1*^*null/+*^ males or *Ksp-*Cre;*Fgfr1*^null/+^ males to obtain the kidney-specific deletion of *Fgfr1* in proximal tubule or distal tubule of these mice. For the entire study, samples were collected from 4-month-old *Fgfr1*^*flox/+*^ (control equivalent) control, conditional *γGT-*Cre;*Fgfr1*^null/flox^ (*Fgfr1*^PT-cKO^)-null mice or *Ksp-*Cre;*Fgfr1*^null/flox^ (*Fgfr1*^DT-cKO^)-null mice, respectively. Tail clips were collected to genotype the mice. REDExtract-N-Amp Tissue PCR Kit (Sigma-Aldrich, St. Louis, MO, USA) was used for DNA extraction and PCR amplification. Mice were genotyped for the *Fgfr1*^*flox*^ allele using forward primer 5′-CTG GTA TCC TGT GCC TAT C-3′ and reverse primer 5′-CCA ATC TGA TCC CAA GAC CAC-3′ (325 bp product for the Fgfr1+ control allele, 400 bp product for the Fgfr1flox floxed allele), and for the Fgfr1null allele using forward primer 5′-GTA TTG CTG GCC CAC TGT TC-3′ and reverse primer 5′-CCA ATC TGA TCC CAA GAC CAC-3′ (300 bp product for the *Fgfr1*^null^ null allele). We collected 12 h urine from control, *Fgfr1*^PT-cKO^ and *Fgfr1*^DT-cKO^ mice. We also collected 12 h urine from control, *Fgfr1*^PT-cKO^, and *Fgfr1*^*DT-cKO*^ mice injected with vehicle (PBS) or rFGF-23 (100 ng/g/mouse). Mice were euthanized by exposing overdose of isoflurane followed by cervical dislocation. Power calculations to determine minimal sample size were performed as previously described [29]. All animal procedures are approved by The University of Tennessee Health Science Center's Animal Care and Use Committee (Protocol number: 12–168.0).

### Serum and urine biochemistry

Blood samples were collected by retro-orbital bleeding before the endpoint and by intracardiac exsanguinations at the endpoint. Urine samples were collected overnight (from 6:00 pm to 6:00 am) in mice housed in metabolic cages. Calcium was measured using a Calcium CPC Liquicolor Kit (Stanbio Laboratories, Boerne, TX, USA) and phosphorus was measured using the phosphomolybdylate-ascorbic acid method. Serum parathyroid hormone (PTH) levels were measured using the Mouse Intact PTH ELISA kit (Immutopics, Carlsbad, CA, USA). Urea nitrogen (BUN), and creatinine were measured using individual kits from Stanbio laboratories. Serum 1,25 dihydroxy vitamin D (1,25(OH)_2_D) was measured using a ELISA kit from MyBioSource.com. Serum full-length fibroblastic growth factor 23 (FGF-23) levels were measured using the FGF-23 ELISA kit (Kainos Laboratories, Tokyo, Japan).

### Immunohistochemistry

After fixation in 10% formalin neutral buffered solution immediately after sacrifice, kidneys were embedded in paraffin. Immunohistochemistry was performed using an UltraSensitive ABC Rabbit IgG staining kit and following the manufacturer’s specifications (ThermoScientific, Rockford, IL). Samples were rehydrated in decreasing ethanol solutions, then immersed in phosphate buffered saline for 10 min followed by quenching in 3% hydrogen peroxide. Slides were blocked for 30 minutes in the kit's blocking solution. Primary antibodies [Klotho antibody, 1:100 from R&D Laboratory, Minneapolis, MN; FGFR1 antibody, 1:100 from Abcam, Cambridge, MA; Npt2a antibody, 1:50 from Alpha Diagnostic International, San Antonio, TX [30], TRPV5 antibody 1:1000 from Alomone Labs, Jerusalem, Israel) were applied to slides and incubated for an hour. Secondary horseradish peroxidase (HRP)-conjugated antibodies were applied following another PBS wash and incubated for 1 hour as indicated. Slides were rewashed for 10 min with PBS, and then the ABC reagent was applied for 30 minutes. Immunostaining was detected using a Metal Enhanced DAB Substrate Kit (Thermo Scientific, Rockford, IL). For Klotho and TRPV5 staining, the secondary antibody was Alexa Fluor 568 1:1000, or 488 1:1000 dye, respectively (Thermo Scientific). Controls were performed by omitting primary antibodies. DAPI was used for counterstaining of the kidney sections.

### Von Kossa and Alizarin Red S staining

Kidney sections of mice were stained for calcium deposition using a Von Kossa Stain kit following manufactures’ instructions (American Mastertech Scientific, Lodi, CA). Alizarin Red S staining was performed as described previously [31].

### High resolution 3D microtomography

The femurs were collected, fixed and dehydrated in 70% ethanol. High-resolution micro-Computed Tomography (μCT40, Scanco Medical, Basserdorf, Switzerland) was used to scan and evaluate the metaphyseal trabecular bone microarchitecture and the midshaft cortical bone parameters. The entire femurs and tibia were scanned in a 12.3 mm diameter sample holder at 6 μm resolution: energy level of 55 KeV and intensity of 145 μA. The trabecular bone volume (mm^3^) and bone volume fraction (BV/TV) was measured within the secondary spongiosa on a set of 50 sections (0.6 mm) underneath the growth plate at a threshold of 200 as previously described [32]. The cortical bone thickness (CtTh, mm) was analyzed from 100 sections chosen at the midshaft of each femur at a threshold of 350.

### Kidney RNA isolation and real-time reverse transcriptase (RT)-qPCR

Total RNA was isolated from whole kidney of mouse at 4 months of age using an RNeasy Mini Kit (Qiagen, Germany). For quantitative real-time RT-PCR, 1.0 μg total RNA isolated from kidney of three genotypes mice was reverse transcribed using an iScript cDNA synthesis kit (Bio-Rad, Hercules, CA, USA) by following the manufacturer’s instructions. PCR reactions contained 1 μl of cDNA (equivalent to 50 ng of total RNA), 300 nM each primers, and 1× iQ SYBR Green supermix (Bio-Rad, Hercules, CA, USA) in a total of 25 μl reaction volume performed with CFX96 Real-Time PCR Detection Systems (Bio-Rad). Relative expression values were evaluated with the 2^-ΔΔ^Ct method using GAPDH as housekeeping gene (forward primer: 5’-CACCACCAACTGCTTAGCC -3’, and reverse primer: 5’-TGGCATGGACTGTGG-TCA-3’). CaBP28K: 5’-AACTGACAGAGATGGCCAGGTTA-3’ (forward), 5’-TGAACTCTTTCCCA ACATTTTGAT-3’ (reverse), PMCA1: 5’-CGCCATCTTCTGCACCA-TT-3’ (forward), 5’-CAGCCATTGCTCTATTGAAAGTTC-3’ (reverse), NCX1: 5’-TCC CTACAAA-ACTATTGAAGGCACA-3’ (forward), 5’-TTTCTCATACTCCTCGTCAT-CGATT-3’ (forward), Npt2a: 5’-ATGCTGGCTTTCCTTAC-3’ (forward), 5’-CCACAATGTTCATGCCTTCT-3’ (reverse), Cyp27b1: 5’-ACACTTCGCACAGTTTACG-3’ (forward), 5’-TTAGCAATCCGCAAGCAC-3’ (reverse), Klotho: 5’- AGCGATAGTTA-CAACAAC-3’ (forward), 5’-GCATTCTCTGATATTATAGTC-3’ (reverse), FGFR1: 5’-AACCTCTAACCGCAGAAC-3’ (forward), 5’-GAGACTCCACTTCCACAG-3’ (reverse), Cyp24a1: 5’- TGGGAAGATGATGG-TGACCC-3’ (forward), 5’-ACTGTTCCT-TTGG-GTAGCGT-3’ (reverse), TRPV5:5’- CTTACG-GGTTGAACACCACCA-3’ (forward), 5’-TTGCAGAACCACAGAGCCTCTA-3’ (reverse), TRPV6: 5’-ATCCGCCGCTATGCACA-3’ (forward), 5’-AGTTTTTCTCCTGAGTCTT-TTTCCA-3’ (reverse).

### Western blot analysis

Kidney tissue (~10 mg) was transferred into T-PER tissue protein extraction reagent (Thermo Scientific, Rockford, IL) and 1x protease inhibitor cocktail with 1mM phenylmethylsulfonyl fluoride (PMSF)(Cell Signaling, Danvers, MA, USA). After three 30-second sonication, samples were centrifuged at 13,000x *g* for 10 minute and protein contents in the supernatants were quantified and samples were stored at -80°C until use. Total cell membrane protein was isolated as described previously [12]. For electrophoresis, samples were prepared by mixing 3x SDS loading buffer (Cell signaling) with 1x DTT. About 50 μg of protein were loaded onto NuPAGE 4–12% Bis-Tris Gel (Invitrogen, Carlsbad, CA, USA). Proteins were separated at 150 V for 60 minutes and transferred to nitrocellulose membrane (Invitrogen, Carlsbad, CA). Membranes were blocked with Superblock blocking buffer in TBST (Thermo Scientific, Rockford, IL, USA) for 30 minutes and then incubated with primary antibodies (Klotho antibody, 1:500, from Gwen King’s lab; FGFR1 antibody 1:1000, Cell Signaling Technology, Danvers, MA; TRPV5 1:1000 from Santa Cruz Biotechnology, Dallas, TX; and, Npt2a 1:1000, Alpha Diagnostic International, San Antonio, TX [33]) with gentle agitation overnight at 4°C. After 3 washes with TBST (15 min once and 2x 5 min), membrane was incubated with secondary antibody in Superblock blocking buffer at room temperature for 1 hour. Membrane was then washed 4 times (15 min and 3x 5 min) and subjected to ECL (Thermo Scientific) and analyzed with the FOTO/Analyst Luminary/FX imaging workstation (FOTODYNE INCORPORATED, Hartland, WI, USA). The intensity of bands was quantified using Image J software (http://rsb.info.nih.gov/ij/).

### Statistics

We evaluated differences between two groups by unpaired t-test and multiple groups by one-way ANOVA followed by Tukey’s post-test for multiple comparisons. All values are expressed as means ± SD. All statistical tests are performed with an alpha of 0.05 as the significance threshold. All computations were performed using GraphPad Prism5 (GraphPad Software Inc. La Jolla, CA, USA).

## Results

### Proximal and distal tubule specific deletion of *Fgfr1* in the kidney

**Fig 1:**
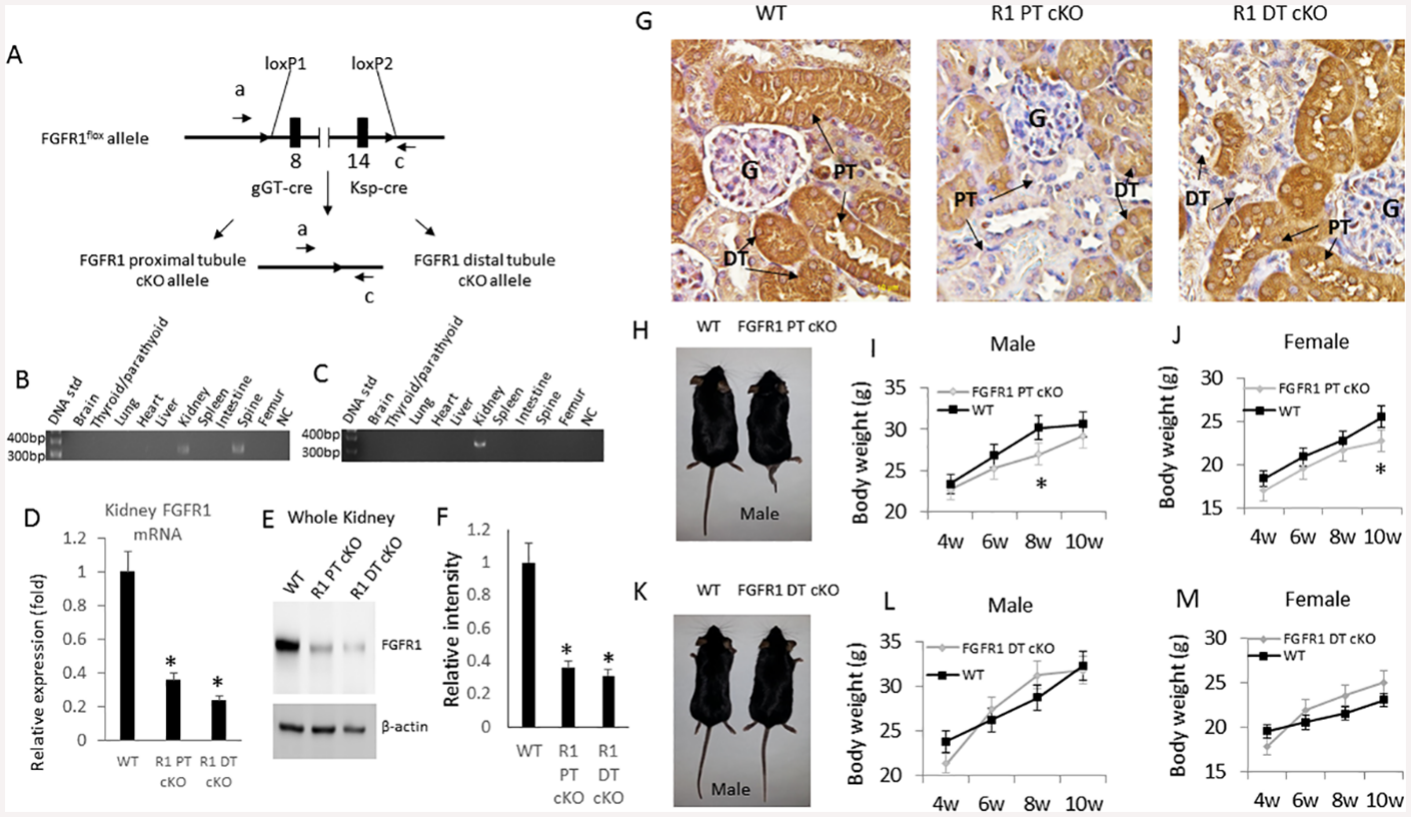
Generation of FGFR1 conditional knockout in the distal tubule of mouse kidney. (A) Schematic deletion of FGFR1 gene in proximal tubule or distal tubule of kidney by crossing *FGFR1*^*flox/flox*^ female mouse with *γGT-Cre;Fgfr1*^*null/+*^ or *Ksp-Cre;Fgfr1*^*null/+*^ male mouse. (B) Genotyping of *Fgfr1*^PT-cKO^ mouse. (C) Genotyping of *Fgfr1*^*DT-cKO*^ mouse. (D) Expression of FGFR1 mRNA in the kidney of control, *Fgfr1*^PT-cKO^, and *Fgfr1*^DT-cKO^ mice as determined by real-time PCR. (E) Western blot analysis of expression of FGFR1 in the whole kidney of control, *Fgfr1*^*PT-cKO*^ or *Fgfr1*^DT-cKO^ mice. (F) Quantitation of FGFR1 Western blot. Expression of β-actin was used as internal controls. (G) Immunohistochemical staining of FGFR1 in the kidney of control, *Fgfr1*^PT-cKO^, and *Fgfr1*^DT-cKO^ mice. Letter G indicates glomeruli, and PT and DT indicate proximal and distal tubules, which have distinct morphological features. Magnification 400X. (H) Photo image of control and *Fgfr1*^PT-cKO^ mice. (I) Growth charts of *Fgfr1*^PT-cKO^ male and female (J). K. Photo image of control and *Fgfr1*^DT-cKO^ mice. (L) Growth charts of *Fgfr1*^DT-cKO^ male and female (M). Data were collected from mice (n = 5) of each genotype. One-tail unpaired *t* test *P<0.05 vs control.

Gamma-glutamyltransferase-Cre (*γGT-cre*) and kidney specific-Cre (*Ksp*-Cre) to conditionally delete *Fgfr1* in proximal tubule or distal tubule of the kidney successfully created *Fgfr1*^PT-cKO^ and *Fgfr1*^DT-cKO^ mice (Fig 1A), respectively. Both *Fgfr1*^PT-cKO^ mice and *Fgfr1*^*DT-cKO*^ mice were born with the expected Mendelian frequency. There were no differences in survival between the genotypes produced by this breeding strategy. All mice survived to adulthood.

To confirm γGT-Cre and Ksp-Cre mediated recombination occurred selectively in the kidney we performed PCR analysis of genomic DNA using primer sets that specifically detect the excised flox alleles. We found γGT-Cre and Ksp-Cre excision of the *Fgfr1* floxed alleles occurred in the kidney in both *Fgfr1*^PT-cKO^ mice (Fig 1B) and *Fgfr1*^DT-cKO^ mice (Fig 1C). We found no evidence of the excised flox alleles in a wide variety of other tissues tested using Ksp-Cre, but found expression of γGT-Cre in a sample obtained from spine but not long bone.

Expression of *Ffgr1* message and protein levels were reduced by ~75% in the kidney of both *Fgfr1*^PT-cKO^ and *Fgfr1*^DT-cKO^ mice as determined by qRT-PCR (Fig 1D) and Western blot analysis (Fig 1E and 1F). We confirmed the specific deletion of FGFR1 in the proximal tubule of *Fgfr1*^PT-cKO^ mice and in the distal tubule of *Fgfr1*^DT-cKO^ mouse by immunohistochemical staining with an anti-FGFR1 antibody (Fig 1G). FGFR1 immuno-reactivity was detected in both distal and proximal tubules in control mice (Fig 1G), but FGFR1 expression was selectively reduced in the morphologically distinct proximal tubular segments in *Fgfr1*^PT-cKO^ mice and in the distal tubular segments in *Fgfr1*^DT-cKO^ mice, respectively (Fig 1G). Differences in FGFR1 expression in morphologically distinct proximal and distal tubular segments are apparent in γGT-Cre and Ksp-Cre conditional knockout mice (S1 Fig). Indeed, FGFR1 staining was only seen in the distal tubules of *Fgfr1*^PT-cKO^ mice and in the proximal tubules of *Fgfr1*^DT-cKO^ mice (Fig 1G and S1 Fig). FGFR1 staining was most predominant on the luminal membranes S1 Fig).

*Fgfr1*^PT-cKO^ mice exhibited gross skeletal abnormalities characterized by curled tails, smaller body size and reductions in body weight (Fig 1H, 1I and 1J). *Fgfr1*^DT-cKO^ mice exhibited no gross abnormalities and body weight no different from control mice (Fig 1K, 1L and 1M).

### Serum biochemistries in mice with proximal and distal tubule deletion of *Fgfr1*

**Fig 2:**
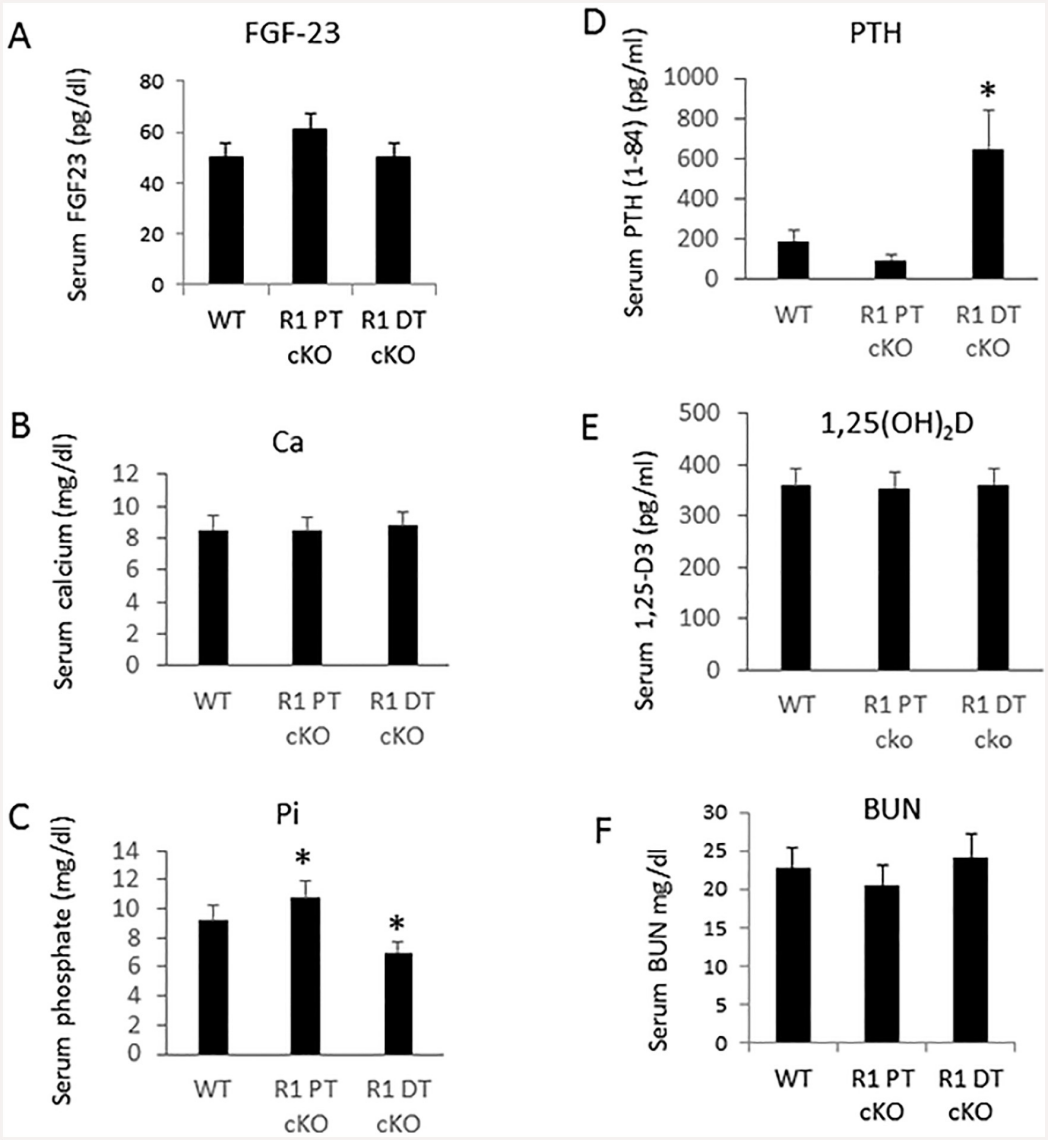
Conditional knockout of distal tubule FGFR1 results in elevated serum PTH. FGF-23 (A), calcium (B), phosphate (C), PTH (D), 1,25(OH)_2_D (E), blood urea nitrogen (BUN) (F) were measured in serum samples collected from 16-weeks-old control, *Fgfr1*^PT-cKO^, or *Fgfr1*^DT-cKO^ mice. Data were collected from mice (n = 5) of each genotype. One-tail unpaired *t* test *P<0.05 vs control.

We observed no significant differences in circulating concentrations of FGF-23, calcium, or 1,25(OH)_2_D, between control, *Fgfr1*^PT-cKO^, and *Fgfr1*^DT-cKO^ mice under basal conditions (Fig 2A, 2B and 2E). Serum BUN were not different between *Fgfr1*^PT-cKO^, *Fgfr1*^*DT-cKO*^ and control mice (Fig 2F). We found that the level of serum phosphate was significantly higher (p<0.05) in *Fgfr1* ^PT-cKO^ mice and significantly lower (p<0.05) (Fig 2C) in *Fgfr1*^DT-cKO^ compared to controls. In addition, serum PTH levels were similar to controls in *Fgfr1* ^PT-cKO^ mice, but significantly increased in *Fgfr1*^DT-cKO^ mice (Fig 2D).

**Fig 3:**
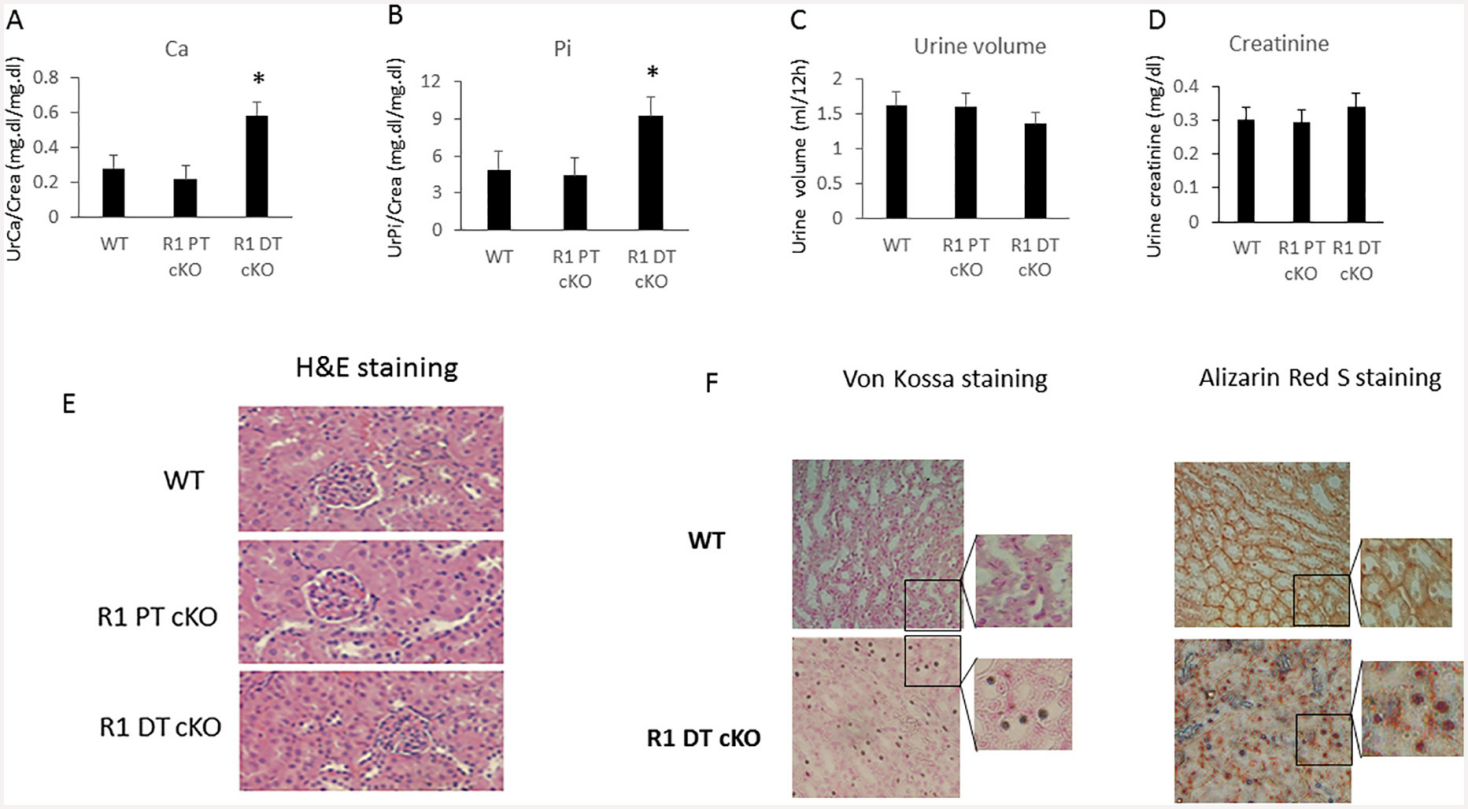
*Fgfr1*^DT-cKO^ mice show urine calcium wasting and calcium deposition in the distal tubular of kidney. Urinary calcium (A), phosphate (B), volume (C), and creatinine (D) were assessed in a 12h urine collection in 16-weeks-old control, *Fgfr1*^*PT-cKO*^ or *Fgfr1*^DT-cKO^ mice. Kidney sections of control, *Fgfr1*^PT-cKO^, or *Fgfr1*^DT-cKO^ mice. Kidney sections for H&E staining (E), magnification 200X and Von Kossa and Alizarin Red S staining (F), magnification 100X. Date were collected from mice (n = 5–6) of each genotype. One-tail unpaired *t* test *P<0.05 vs control.

Urinary calcium and phosphate excretion were not different between *Fgfr1*^PT-cKO^ and controls; however, both urinary calcium and phosphate excretion were significantly higher in *Fgfr1*^DT-cKO^ mice than control mice (p<0.05) (Fig 3A and 3B). Urine volume and urine creatinine levels were similar in control, *Fgfr1*^PT-cKO,^ and *Fgfr1*^*DT-cKO*^ mice (Fig 3C and 3D). Both *Fgfr1*^PT-cKO^ and *Fgfr1*^*DT-cKO*^ had normal renal architecture (Fig 3E), indicating that loss of FGFR1 had no effect on kidney development.

### Kidney micro-calcifications and abnormal bone structure in *Fgfr1* ^*PT-cKO*^ and *Fgfr1* ^*DT-cKO*^ mice

Kidney sections from *Fgfr1*^DT-cKO^ mice showed evidence of small dense deposits foci in the tubular lumen mainly in the medullary regions that were positively by both Von Kossa and Alizarin Red staining (Fig 3F). We observed discrete intratubular dense deposits in the distal tubules in *Fgfr1*^DT-cKO^ but not control mice. The intra-luminal deposits were not associated with cell injury or inflammation. We also did not observed soft-tissue calcification of the kidneys by μCT analysis (data not shown). These unusual appearing structures resembled calcium phosphate microcrystal masses previously observed in renal tubules following intestinal resection in rats [34].

**Fig 4:**
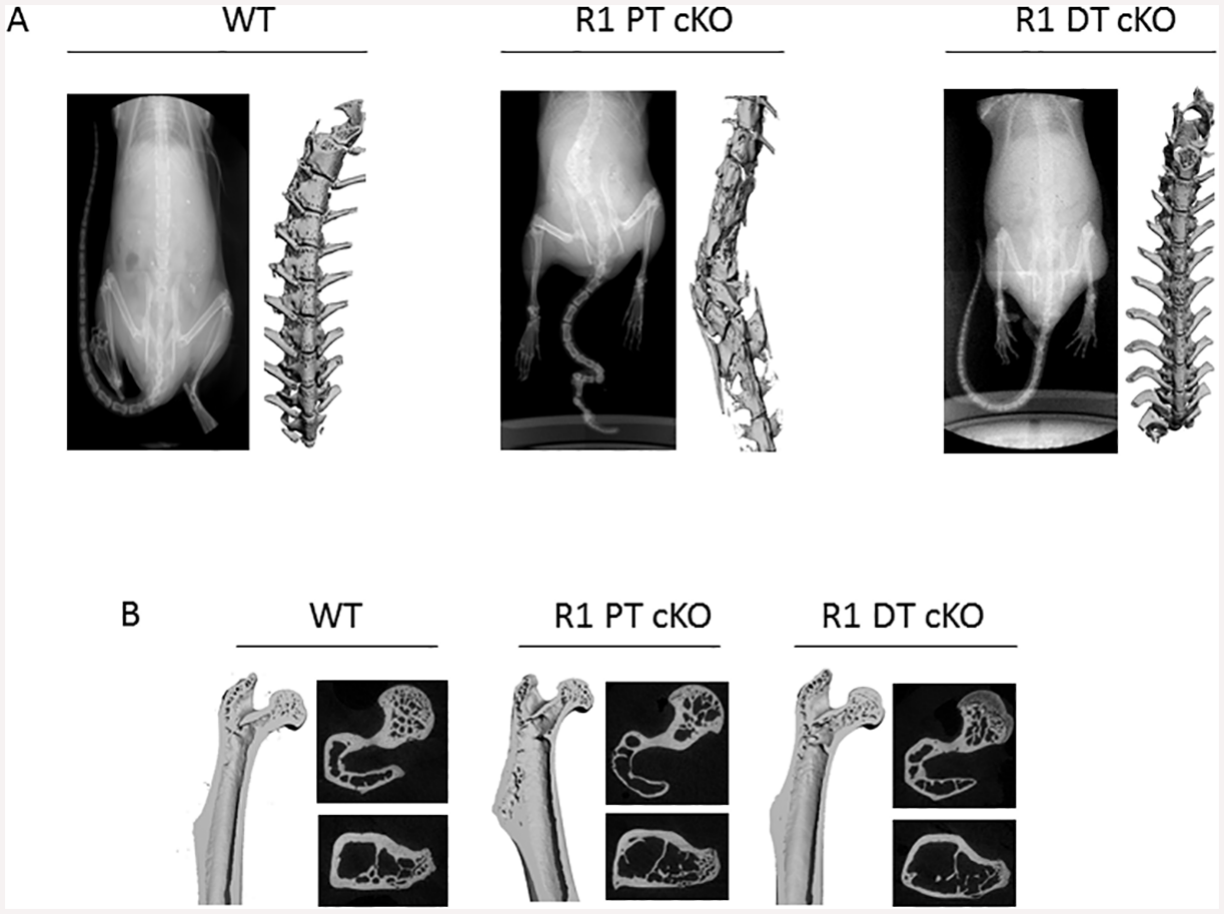
Conditional deletion of FGFR1 resulted in abnormal bone structures in *Fgfr1*^PT-cKO^ and *Fgfr1*^DT-cKO^ mice. (A) X-ray and μCT images of spinal bone of control, *Fgfr1*^PT-cKO^, and *Fgfr1*^DT-cKO^ mice. (B) μCT images of long bone from control, *Fgfr1*^PT-cKO^, and *Fgfr1*^DT-cKO^ mice. Images are representative of samples collected from mice (n = 5) of each genotype.

We further characterized the skeletal abnormalities in *Fgfr1*^PT-cKO^ mice by X-ray and μCT analysis (Fig 4A). The radiograph and three dimensional spine images demonstrated that the spinal deformities of vertebrae. The involved vertebral bodies lost normal anatomic structures with irregular wedge-shaped morphology or fusion of several segments. Curly tails and vertebral deformities were observed in ~ 88% and ~44% of *Fgfr1*^PT-cKO^ mice, indicating incomplete penetrance. Since the spinal defect resembles spina bifida, which can be caused by folic acid deficiency, we measured serum folic acid in *Fgfr1*^PT-cKO^ mice. Serum folic acid concentrations (mean± SEM, ng/ml) were not different between *Fgfr1*^PT-cKO^ (11.64±2.63), *Fgfr1*^DT-cKO^ (10.72±0.88), and control (11.04±1.18) mice. In addition to the skeletal deformities of the spine, we found that both trabecular bone volume and trabecular bone fraction of femoral bone were significantly decreased in *Fgfr1*^PT-cKO^ mice (Table 1 and Fig 4B). In contrast, *Fgfr1*^DT-cKO^ mice exhibited no gross abnormalities (Fig 1K). However, trabecular thickness, trabecular material, and cortical thickness of femur bone was significantly reduced in *Fgfr1*^DT-cKO^ mice compared to c006Fntrols (Table 1 and Fig 4B).

**Table 1:** Micro CT analysis of femur bone structure changes in control, *Fgfr1*^PT-cKO^, and *Fgfr1*^DT-cKO^ mice.

Femur	Control	*Fgfr1*^PT-cKO^	Control	*Fgfr1*^DT-cKO^
Trabecular Bone Volume (mm3)	0.328 ± 0.019	0.1863 ± 0.093*	0.273 ± 0.145	0.235 ± 0.056
Trabecular Bone Fraction (BV/TV)	0.177 ± 0.017	0.101 ± 0.052*	0.156 ± 0.075	0.131 ± 0.033
Trabecular Number (1/mm)	3.813 ± 0.156	3.428 ± 0.543	4.302 ± 0.797	4.394 ± 0.567
Trabecular Thickness (mm)	0.049 ± 0.004	0.043 ± 0.01	0.050 ± 0.006	0.043 ± 0.002*
Trabecular Material BMD (mgHA/mm^3)^	913.1 ± 21.3	896.1 ± 28.2	896.6 ± 19.2	859.7 ± 8.9**
Cortical Thickness (mm)	0.204 ± 0.011	0.203 ± 0.006	0.196 ± 0.012	0.179 ± 0.013*

Data were presented as mean ± S.D. from mice (n = 5) of each genotype. One-tail unpaired *t* test

*P<0.05,

**P<0.01 vs control.

### Effect of *Fgfr1* conditional deletion on proximal and distal tubule gene expression

**Fig 5:**
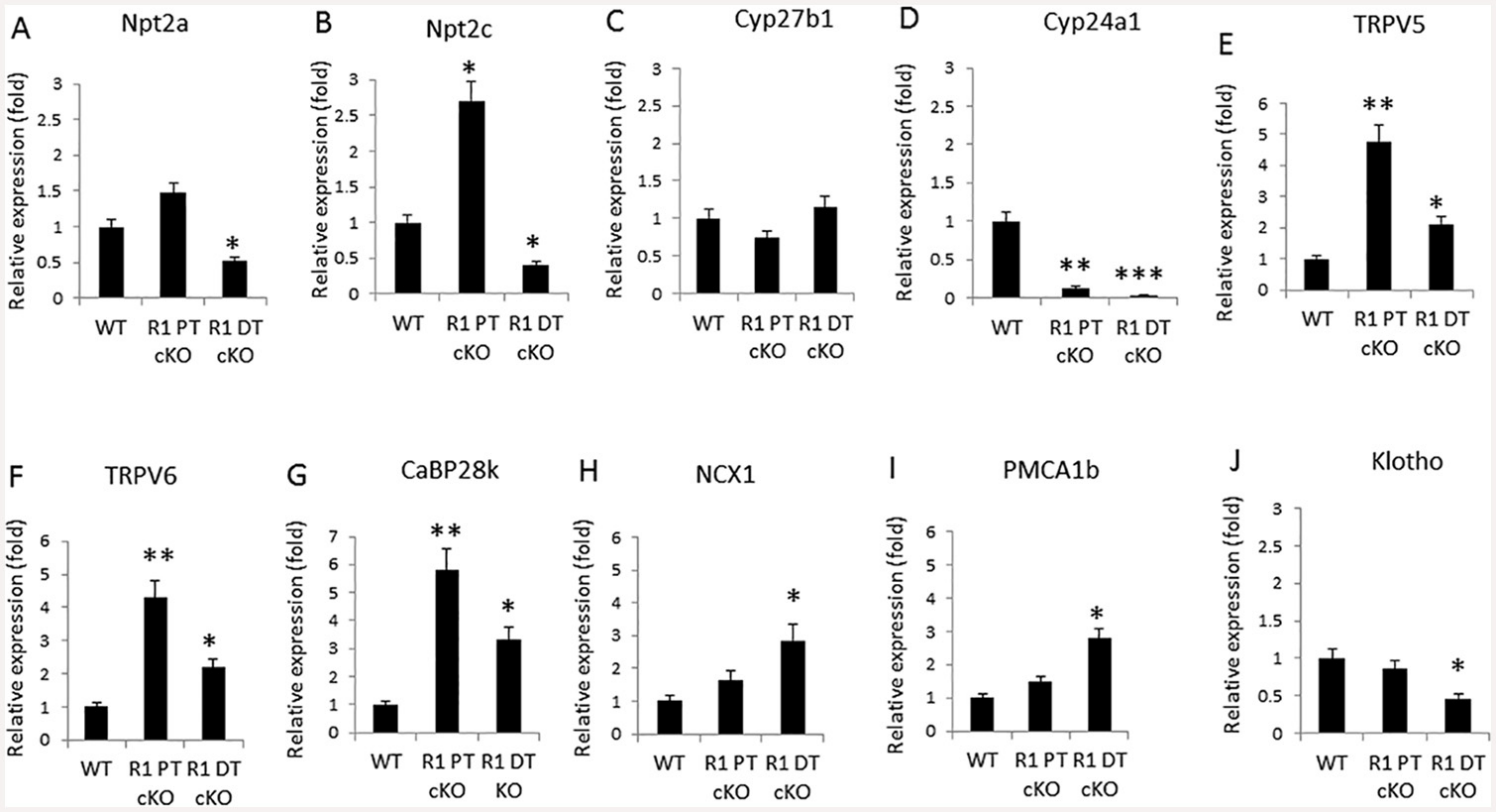
Upregulation of calcium transport related genes in *Fgfr1*^PT-cKO^ and *Fgfr1*^DT-cKO^ mice. Gene expression in kidney of 16-weeks-old control, *Fgfr1*^PT-cKO^_,_ and *Fgfr1*^DT-cKO^ mice was determined by real-time PCR as follows: Npt2a (A), Npt2c (B), Cyp27b1 (C), Cyp24a1 (D), TRPV5 (E), TRPV6 (F), CaBP28k (G), NCX1 (H), PMCA1b (I), and α-Klotho (J). Data represented mean ± S.D. of kidney samples (n = 5) from mice of each genotype. One-tail unpaired *t* test *P<0.*05*, **P<0.*01*, ***P<0.*001* vs control.

Next, we examined the effects of proximal tubule deletion of *Fgfr1* on renal tubular transporters involved phosphate transport and vitamin D metabolism (Fig 5). In *Fgfr1*^PT-cKO^ mice, messenger RNA levels for the sodium phosphate cotransporters *Npt2a* and *Npt2c* were increased 1.5-fold and 2.5-fold, respectively (Fig 5A and 5B); but only expression of *Npt2c* achieved significance compared to controls (p<0.05). Expression of 25-hydroxyvitamin D-1 alpha hydroxylase (*Cyp27b1*) was not affected, but expression of *Cyp24a1* was significantly reduced (p<0.01) in *Fgfr1*^*PT-cKO*^ mice compared to controls (Fig 5C and 5D). In spite of observing no alterations in urinary calcium compared to controls in *Fgfr1*^*PT-cKO*^ mice, loss-of-*Fgfr1* in the PT resulted in significant increments message levels for transient receptor potential cation channel subfamily V member 5 (*TRPV5*), *TRPV6*, and calcium binding protein 28k (*CaBP28k*) in the kidney (p<0.05) (Fig 5E, 5F, and 5G). Levels of sodium-calcium exchanger (*NCX1*), plasma membrane calcium pump-1b (*PMCA1b*), and *α-Kl* transcripts, however, were not changed in *Fgfr1*^PT-cKO^ mice compared to control (Fig 5H, 5I, and 5J).

In *Fgfr1*^DT-cKO^ mice, the expression of sodium phosphate cotransporters mRNA, *Npt2a* and *Npt2c*, were both significantly decreased compared to controls (p<0.05) (Fig 5A and 5B). Although expression of *Cyp27b1* was not affected, expression of *Cyp24a1* was significantly reduced in *Fgfr1*^*DT-cKO*^ mice compared to controls (p<0.01) (Fig 5C and 5D). Since PTH suppresses sodium phosphate transporters and inhibits *Cyp24a1*, the observed changes in gene expression are most likely indirectly due to the effects of elevated serum PTH on the proximal tubule.

We also examined the effects of distal tubule deletion of FGFR1 on renal tubular transporters involved in calcium transport (Fig 5E–5J). In spite of the increased urinary calcium, we found that expression of messenger RNA levels for *TRPV5*, *TRPV6*, *CaBP28k*, *NCX1*, and *PMCA1b* were significantly increased in *Fgfr1*^DT-cKO^ compared to control mice (p<0.05) (Fig 5E–5I). In addition, *α-Kl* transcripts were significantly decreased in *Fgfr1*^DT-cKO^ kidneys compared to controls.

### Expression of TRPV5 and α-KL proteins

**Fig 6:**
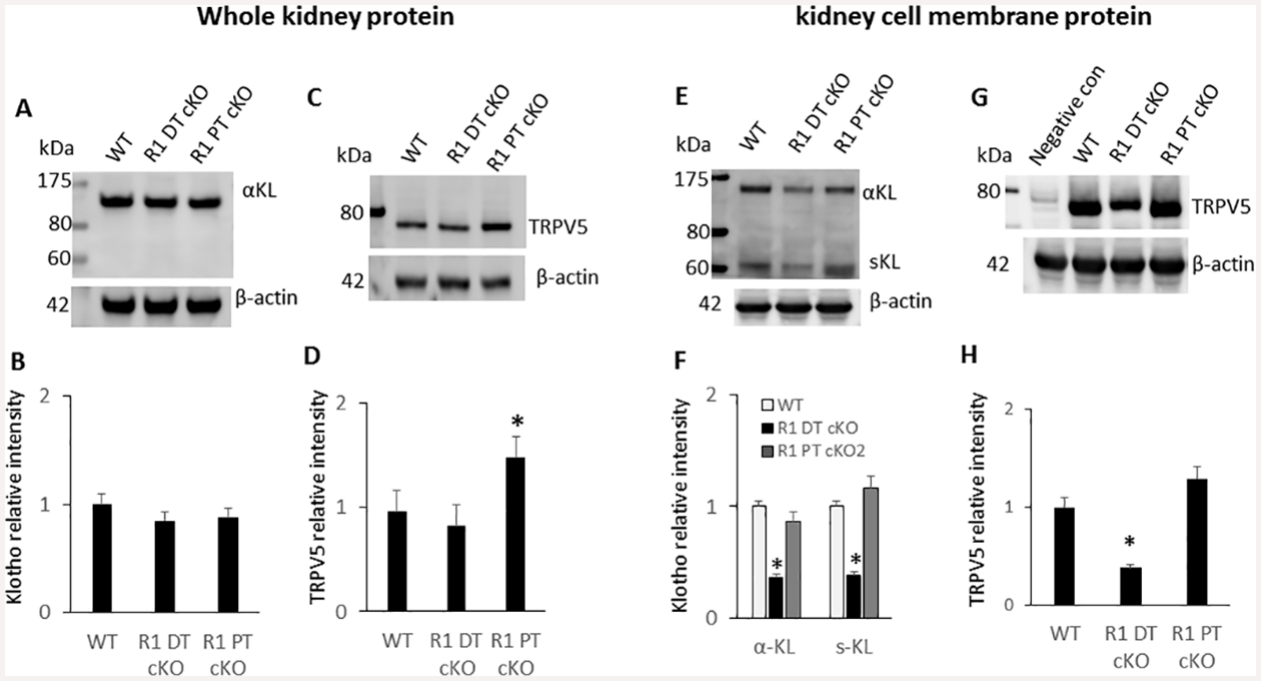
Expression of TRPV5 and α-Klotho in *Fgfr1*^PT-cKO^ and *Fgfr1*^DT-cKO^ mice. Western blot analysis of expression of Klotho and TRPV5 from proteins prepared from the whole kidney or from kidney cortex tissues of 16-weeks-old control, *Fgfr1*^PT-cKO^, or *Fgfr1*^DT-cKO^ mice. (A) Klotho expression detected in whole kidney protein. (B) Quantitation of Klotho by Western blot analysis. (C) TRPV5 expression in whole kidney protein samples. (D) Quantitation of TRPV5 expression from Western blot analysis. (E) Klotho expression in membrane protein. (F) Quantitation of Klotho expression by Western blot analysis. (G) TRPV5 expression in membrane protein isolated from the cortical region of the kidney. Membrane protein isolated from the medulla of the kidney was used as negative control. (H) Quantitation of TRPV5 expression from Western blot analysis. Data represented mean ± S.D. of kidney samples (n = 6) from mice of each genotype. One-tail unpaired *t* test *P<0.05 vs control.

We evaluated the expression of α-Klotho (α-KL) and TRPV5 using proteins isolated from whole kidney and kidney cell membrane. We found that expression of α-KL in the whole kidney protein was not affected in *Fgfr1*^PT-cKO^ or *Fgfr1*^DT-cKO^ mice (Fig 6A and 6B). The increase in TRPV5 message expression observed in *Fgfr1*^PT-cKO^ was associated with only a slight increase in TRPV5 protein by Western blot analysis (Fig 6C and 6D). Interestingly, we found that expression of both Klotho isoforms (α-KL and s-KL) and TRPV5 in isolated kidney membrane proteins were significantly (p<0.05) decreased in *Fgfr1*^DT-cKO^ mice as compared to that of WT or *Fgfr1*^PT-cKO^ mice, respectively (Fig 6E, 6F, 6G, and 6H). Down-regulation of TRPV5 in membrane derived kidney protein from *Fgfr1*^DT-cKO^ mice was also confirmed using a different anti-TRPV5 antibody (S2 Fig).

**Fig 7:**
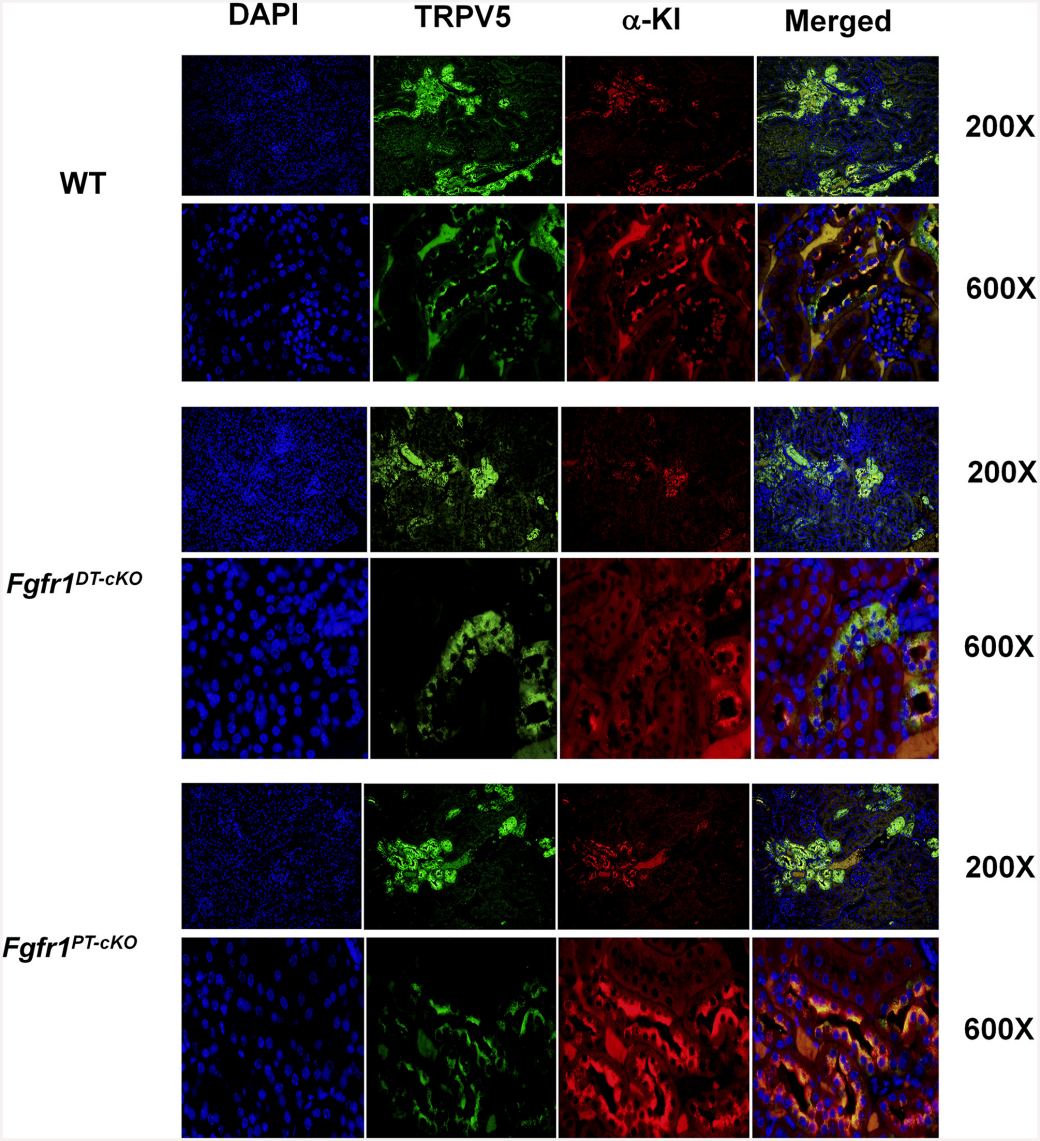
Immunohistochemical staining of TRPV5 and α-Klotho. Kidney sections from 16-weeks-old control, *Fgfr1*^PT-cKO^, or *Fgfr1*^DT-cKO^ mice were stained with TRPV5 or Klotho antibodies and counterstained with DAPI. Magnification of the images are 200X or 600X as indicated. Images data represented kidney of mice (n = 5) from each genotype.

TRPV5, a marker for the distal tubule, was detected only in the distal tubule of control, *Fgfr1*^*PT-cKO*^, and *Fgfr1*^*DT-cKO*^ mouse kidneys by immunohistochemical staining (Fig 7). The distal tubules that express TRPV5 also had morphological features distinct from the proximal tubules exhibited brush borders and lacked expression of TRPV5. α-KL immunoreactivity was detected in the distal tubules of control, *Fgfr1*^*PT-cKO*^, and *Fgfr1*^*DT-cKO*^ mice (Fig 7). α-KL immunoreactivity co-localized with TRPV5 in the distal tubule. We observed no appreciable staining for TRPV5 or Klotho in morphologically distinct proximal tubules characterized by brush border membranes. High power images demonstrated localization of TRPV5 and Klotho to the apical membranes of the distal tubules (Fig 7, lower panels with 600X magnification). Both TRPV5 and α-KL were decreased in *Fgfr1*^*DT-cKO*^. Expression of TRPV5 and α-KL in *Fgfr1*^*PT-cKO*^ was similar to wild-type mice.

### Expression of Npt2a protein

**Fig 8:**
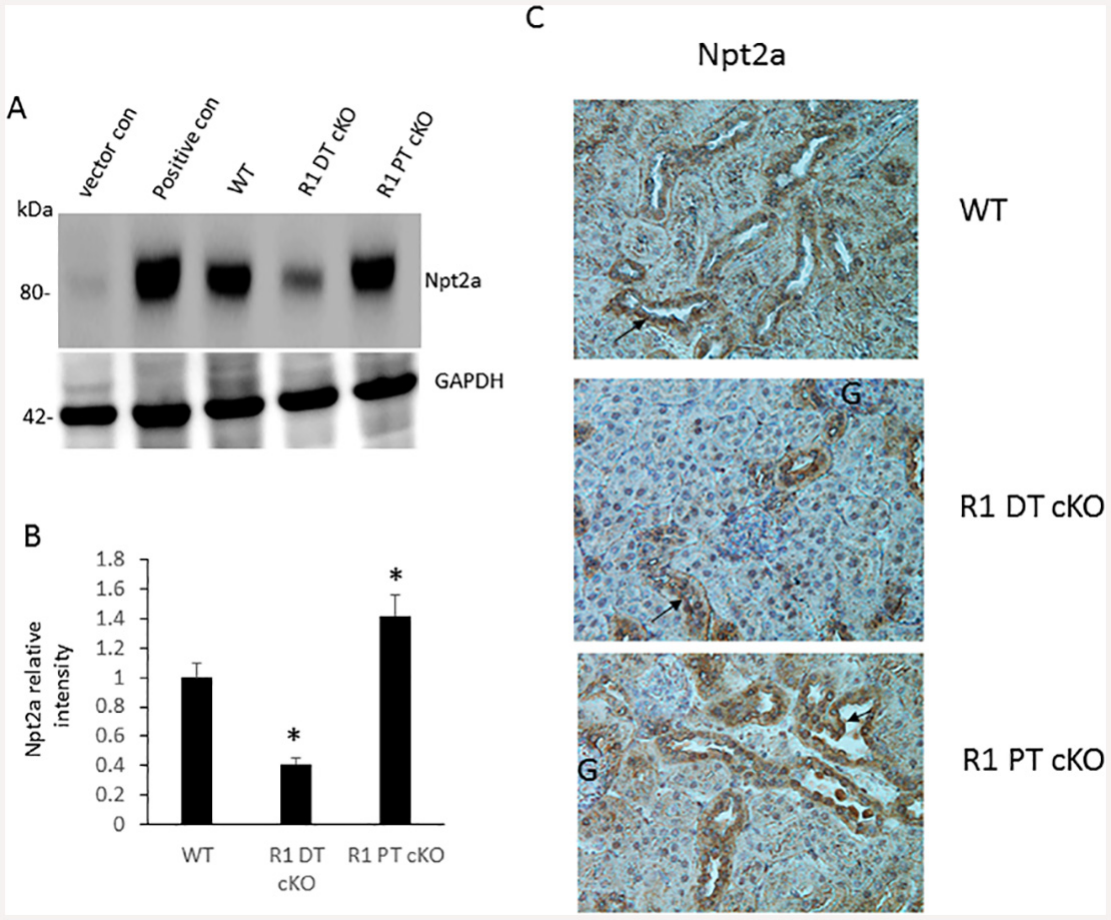
Expression of Npt2a in *Fgfr1*^PT-cKO^ and *Fgfr1*^DT-cKO^ mice. Western blot analysis of expression of Npt2a from membrane proteins prepared from the kidney cortex of 16-weeks-old control, *Fgfr1*^PT-cKO^, or *Fgfr1*^DT-cKO^ mice. (A) Western blot analysis of Npt2a expression. (B) Quantitation of Npt2a protein expression by Western blot analysis. (C) Immunohistochemical staining of Npt2a expression in kidney sections (arrow). The letter G indicates glomeruli. Protein samples isolated from pcDNA3.1 vector-transfected MDCK cells (ATCC) were used as negative control for Npt2a expression [35]. Protein samples isolated from MDCK cells transfected with Npt2a cDNA were used as positive controls. Data represent the mean ± S.D. of kidney samples derived from (n = 6) mice of each genotype. Images are representative of the analysis of five mice from each genotype. Magnification is 400X. One-tail unpaired *t* test *P<0.05 vs controls.

We further examined the expression of sodium-depended phosphate co-transporter gene Npt2a in the kidney of control, *Fgfr1*^PT-cKO^, and *Fgfr1*^DT-cKO^ mice. Western blot analysis of membrane protein showed that expression of Npt2a was significantly downregulated in *Fgfr1*^DT-cKO^ mice (Fig 8A and 8B, and S3 Fig). In contrast, expression of Npt2a protein was significantly upregulated in *Fgfr1*^PT-cKO^ mice (Fig 8A and 8B, and S3 Fig). Immunohistochemical staining of kidney sections showed that Npt2a was located at apical brush border membrane of the proximal tubule (Fig 8C). We showed that staining of Npt2a was increased in *Fgfr1*^PT-cKO^ mice, and decreased in *Fgfr1*^DT-cKO^ mice, respectively (Fig 8C). Thus, the increase in Npt2a protein expression was greater than the increase in Npt2a message expression. Because of the lack of a validated anti-Npt2c antibody, we were unable to assess the effects on Npt2c protein expression.

### Effects of rFGF-23 in *Fgfr1*^*PT-cKO*^ and *Fgfr1*^*DT-cKO*^ mice

Finally, we examined the effect of selective loss of FGFR1 in the distal tubule on the renal response to FGF-23 administration. Administration rFGF-23 at 100 ng/g IP to 16 week-old controls resulted in a 2-fold increase in circulating FGF-23 concentrations 12 hours after injection. rFGF-23 resulted in a significant 32% reduction in serum phosphate concentrations, a 4-fold increase in urinary phosphate excretion, and a 7-fold reduction in *Npt2a* message expression in control mice. rFGF-23 administration had no significant effects of serum calcium, PTH or 1,25(OH)_2_D levels or urinary calcium excretion in control mice. rFGF-23 also suppressed *Cyp27b1*, *α-Kl* (4-fold), and *Fgfr1* (2-fold) message expression in control mice. rFGF-23 treatment resulted in non-significant increments in *TRPV5*, *TRPV6* and *CaBP28k*.

We observed marked differences in proximal tubular responses to rFGF-23 in *Fgfr1*^PT-cKO^ and *Fgfr1*^DT-cKO^ mice. Administration of rFGF-23 resulted in a 2.5 fold increase in serum FGF-23 concentrations 12 hours after dosing in *Fgfr1*^PT-cKO^ mice. rFGF-23 did not reduce serum phosphate in *Fgfr1*^PT-cKO^ mice, but resulted in a ~ 20% increase urinary phosphate excretion, likely due to residual FGFR function. The magnitude of the phosphaturic effect of FGF-23 in *Fgfr1*^PT-cKO^ mice, however, was much lower than observed in controls. In addition, rFGF-23 resulted in an ~50% reduction in *Npt2a* and *Npt2c* expression, but again, the magnitude of this reduction was less that observed in controls treated with rFGF-23.

In *Fgfr1*^DT-cKO^ mice, rFGF-23 administration did not affect serum calcium, 1,25(OH)_2_D or PTH levels. Moreover, the proximal tubule actions of FGF-23 were not inhibited by distal tubule deletion of FGFR1. Indeed, rFGF-23 significantly reduced the already lowered serum phosphate levels further (from 6.98 to 6.29 mg/dl), increased the urinary phosphate excretion (from 9.27 to 13.63 Pi/Cr), and markedly reduced the expression of *Npt2a* and *Npt2c* message expression in *Fgfr1*^DT-cKO^ mice (Table 2). rFGF-23 also suppressed *Cyp27b1* in *Fgfr1*^DT-cKO^ mice.

**Table 2:** Serum and Urine Biochemistries and Gene Expression in *Fgfr1*^PT-cKO^ and *Fgfr1*^DT-cKO^ before and after rFGF-23 administration.

	Wild type		*Fgfr1*^PT-cKO^		*Fgfr1*^DT-cKO^	
**Serum (n = 8)**	Vehicle	rFGF23	Vehicle	rFGF23	Vehicle	rFGF23
FGF23 (pg/dl)	50.3±15.3^a^	93.0±19.6^b^	65.2±18.3^a^	158.9±38.2^b^	50.4±13.2^a^	129.9±30.6^b^
Calcium (mg/dl)	8.54±0.64^a^	8.93±0.66^a^	8.48±0.63^a^	8.69±0.64^a^	8.77±0.59^a^	9.23±0.46^a^
Phosphate (mg/dl)	9.21±0.26^a^	6.23±0.18^b^	10.75±0.33^d^	10.21±0.42^d^	6.98±0.31^c^	6.29±0.22^b^
1,25(OH)2D3 (pg/ml)	359±42^a^	341±45^a^	354±36^a^	357±43^a^	361±35^a^	340±39^a^
PTH (pg/dl)	188±52^a^	194±45^a^	93±36^a^	110±26^a^	647±189^b^	504±181^b^
BUN (mg/dl)	22.7±2.5^a^	26.4±2.9^a^	20.7±3.2^a^	21.4±3.0^a^	24.2±2.6^a^	25.9±3.1^a^
Creatinine (mg/dl)	0.10±0.01^a^	0.09±0.01^a^	0.11±0.03^a^	0.10±0.01^a^	0.09±0.01^a^	0.10±0.01^a^
**Urine (n = 5)**						
Urine volume (ml)	1.61±0.31^a^	1.95±0.36^a^	1.60±0.41^a^	1.80±0.42^a^	1.35±0.26^a^	1.85±0.34^a^
Calcium (Ca/Crea)	0.28±0.05^a^	0.29±0.06^a^	0.22±0.04^a^	0.82±0.19^b^	0.59±0.09^b^	0.71±0.12^b^
Phosphate (Pi/Crea)	3.44±0.86^a^	14.29±2.31^b^	5.09±1.10^a^	6.04±1.12^a^	9.27±1.12^b^	13.63±2.14^b^
**Gene (n = 5)**						
FGFR1	1.00±0.26^a^	0.49±0.09^b^	0.36±0.08^b^	0.22±0.08^c^	0.24±0.04^c^	0.21±0.08^c^
NPT2a	1.00±0.29^a^	0.13±0.11^c^	1.47±0.35^a^	0.97±0.23^a^	0.52±0.05^b^	0.07±0.08^c^
NPT2c	1.00±0.33^a^	0	2.71±0.30^b^	1.56±0.18^c^	0.41±0.10^b^	0
Cyp27b1	1.00±0.28^a^	0.55±0.12^b^	0.74±0.16^a^	0.53±0.13^b^	1.15±0.33^a^	0.5±0.10^b^
TRPV5	1.00±0.32^a^	1.47±0.36^a^	4.76±0.52^b^	2.08±0.50^b^	2.11±0.18^b^	1.24±0.29^a^
TRPV6	1.00±0.12^a^	1.20±0.31^a^	4.30±0.46^b^	1.00±0.34^a^	2.20±0.43^b^	0.91±0.13^a^
CaBP28k	1.00±0.26^a^	1.28±0.29^a^	5.81±0.63^b^	2.56±0.61^b^	3.31±0.51^b^	1.81±0.36^b^
Klotho	1.00±0.21^a^	0.27±0.15^c^	0.86±0.21^a^	0.25±0.16^c^	0.46±0.12^b^	0.20±0.25^c^

Data represent mean ± S.D. from mice (n = 5–8) of each genotype. One-way ANOVA analysis with post hoc test for multiple comparisons. Values not sharing the same superscript letter are significantly different at *P*<0.05.

With regards to distal tubular functions, in *Fgfr1*^PT-cKO^ mice, we found that rFGF-23 increased urinary calcium 4-fold in association with significant reductions in *TRPV5*, *TRPV6* and *α-Kl* expression. In *Fgfr1*^DT-cKO^ mice, rFGF-23 had a slight, but non-significant effect to further increase in urinary calcium excretion. Interestingly, however, high urinary calcium persisted, in spite of the effect of FGF-23 to suppress *TRPV5*, *TRPV6*, and *CaBP28k* transcripts in *Fgfr1*^DT-cKO^ mice, suggesting an uncoupling between transport and gene expression.

## Discussion

Our studies are the first to assess the separate functions of FGFR1 in the proximal and distal tubules of the kidney in mice. By selectively deleting FGFR1 in the proximal tubule using *γGT-Cre*, which expresses Cre recombinase in kidney proximal tubules after nephrogenesis is complete [27], we were able to assess the postnatal and proximal tubule specific functions of this receptor. Reductions of FGFR1 expression in the proximal tubule resulted in hyperphosphatemia and resistance to the phosphaturic effects of administered recombinant FGF-23. These findings are consistent with the known actions of FGF-23 to regulate *Npt2-*mediated phosphate transport, but also suggest that FGFR1 has a greater effect to regulate *Npt2c* message expression. Interestingly, selective loss of FGFR1 in the proximal tubule in *Fgfr1*^*PT-cKO*^ mice did not alter serum 1,25(OH)_2_D levels or *Cyp27b1* expression at baseline or in response to recombinant FGF23 administration, but did reduce *Cyp24a1* expression.

Since FGF-23 activation of FGFRs reduces 1,25(OH)_2_D production in the proximal tubule through suppression of *Cyp27b1* and stimulation of *Cyp24a1*, our findings suggest possible involvement of other FGFRs in mediating renal responses. Indeed, FGFR3 and FGFR4 are expressed in the proximal tubule and have been implicated in mediating FGF-23 effects. In prior studies, we found that compound mutant *Fgfr3*^-/-^/*Fgfr4*^-/-^ mice completely reversed the effects of FGF-23 to suppress 1,25(OH)_2_D, but only partially corrected the renal phosphate wasting [20]. These results, along with current findings in *Fgfr1* ^*PT-cKO*^ mice, suggest that FGFR1, FGFR3 and FGFR4 have overlapping roles to regulate phosphate transport and distinct functions in the proximal tubule to regulate vitamin D metabolism [18, 36]. Although our studies did not examine the down-stream signaling pathways, FGF-23 activation of FGFRs regulates phosphate transport in the proximal tubule through stimulation of extracellular signal-regulated kinase 1 and 2 (ERK1/2) and serum and glucocorticoid-regulated kinase 1 (SGK1) phosphorylation of the Na+/H+ exchange regulatory cofactor 1 (NHERF-1) [23]. How FGFR1, FGFR3 and FGFR4 in the proximal tubule, which have common down-stream signaling pathways, concordantly regulate phosphate transport and differentially regulate vitamin D metabolism remain to be determined.

To our knowledge, no other studies have directly assessed the functions of FGFR1 in the distal tubule. Using *Ksp*-Cre to conditionally delete FGFR1 from the distal tubule, we unexpectedly observed dramatic alterations of both calcium and phosphate homeostasis in mice. Indeed, conditional deletion of FGFR1 in the distal tubule resulted in a marked increase in urinary excretion of calcium that was severe enough to cause secondary hyperparathyroidism. The increase in urinary calcium was accompanied by an increase in urinary phosphate excretion and suppression of Npt2a and Npt2c expression in the proximal tubule in association with significant increments in PTH levels, but no alterations in 1,25(OH)_2_D or changes in circulating FGF-23 levels. In addition, we found evidence for microcrystal formation in the tubular lumen, likely due to the increased calcium and phosphate excretion by the kidney.

Our findings that FGFR1 regulates distal tubular calcium transport are consistent with recent reports showing that FGF-23 regulates calcium reabsorption in the distal nephron through stimulation of ERK/SKG1/WNK4-depedendent trafficking of TRPV5 to the plasma membrane [12]. Since hypercalciuria and secondary hyperparathyroidism were not observed in compound mutant *Fgfr3*^-/-^/*Fgfr4*^-/-^ mice [20], our data suggest that FGFR1 is the principal FGFR regulating calcium transport in the distal tubule. Consistent with this notion, we found that loss of FGFR1 in the distal tubule diminished membrane expression of TRPV5. Indeed, calcium wasting occurred in *Fgfr1*^DT-cKO^ mice in spite of compensatory increases in PTH and increments in expression of genes that would be predicted to increase the transcellular calcium transport in the distal tubule, including calbindin D28K (CaBP28k), the sodium-calcium exchanger (NaCX), PMCA1b, calcium channel transient receptor potential vannilloid-5 and -6 (TRPV5 and TRPV6). FGFR1 effects to diminish membrane expression of TRPV5 in F*gfr1*^DT-cKO^ mice likely accounts for both the inability of PTH and intracellular calcium transport pathways to compensate for the hypercalciuria. Interestingly, we also found that loss of FGFR1 had greater effects of TRPV5 message than protein expression. Similarly, FGFR1 also had greater effects on Npt2a protein than message expression in the proximal tubule. This may be due to known effects of FGFR1 to recruit RNA to polysomes to increase in protein expression in other systems [37]. Nevertheless, a role for FGFR1 in the regulation of renal calcium handling and the activation of this receptor by FGF-23 creates a possible regulatory endocrine loop, since circulating calcium levels are an important regulator of FGF-23 expression and secretion from bone [11, 38].

α-KL is an obligate co-receptor for FGF-23 activation of FGFR1, FGFR3 and FGFR4 in renal tubules [13]. Our findings that FGFR1 mediated FGF-23 functions in the distal tubule are consistent with the observation that α-KL is predominantly expressed in the distal tubule, and *in vivo* pulse chase experiments showing FGF-23 activation of FGFR signaling occurs only in the distal tubule [24, 25]. The role of α-KL in mediating the effects of FGFR1 in the proximal tubule is less certain. Loss of FGFR1 in the proximal tubule blocked FGF-23 phosphaturic effects, in spite of our inability to detect α-KL in the proximal tubule by immunohistochemistry. However, *α-Kl* message is present isolated proximal tubule segments [9], suggesting that functional FGFR/α-KL complexes are present in the proximal tubule. This explanation, however, does not account for the hyperphosphatemia and compensatory increments in serum FGF-23 concentrations in mice with conditional deletion of α-*Kl* in the distal tubule, which appears to support the presence of a distal to proximal tubule feedback mechanism [25]. Indeed, we would have predicted that the conditional deletion of *Fgfr1* and α-*Kl* from the distal tubule using the same *Ksp*-Cre would have result in similar phenotypes. Rather, selective deletion of *Fgfr1* in the distal tubule inhibited renal calcium transport with secondary effects to inhibit proximal tubular phosphate transport, whereas conditional deletion of α-*Kl* in the distal tubule resulted in hyperphosphatemia and no reported effect on renal calcium handling, a phenotype resembling *Fgfr1*^PT-cKO^ mice. Additional studies will be needed to reconcile these discrepancies, but endocytoses by the proximal tubule of α-KL released into the circulation by ectodomain shedding from the distal tubule has been proposed to sequester Npt2a transporters, leading to FGF-23 independent phosphaturia [39]. It is possible, but not tested, that soluble KL uptake in the proximal tubule may also function as a co-factor for FGFR1 activation [39–42].

Finally, the mechanism underlying the observed curly tail and spina bifida-like malformation in *Fgfr* ^*PT-cKO*^ mice are not clear. A similar phenotype has been observed by loss of Fgfr1 in the neural tissues in the posterior portion of the spinal cord in *Fgfr1*^*-/-*^ mice [43]. Although we did not test for FGFR1 deletion the specifically in the developing neural tube of *Fgfr1* ^*PT-cKO*^, the tail distortion and spina bifida in the setting of normal serum folate and normal trabecular and cortical bone development, suggests that the γGT-Cre may be expressed in critical sites controlling spinal development. Importantly, however, the development of other bones were normal, and γGT-Cre was not expressed in long bones. We also observed no reductions in FGF-23, which would be expected if we had deleted FGFR1 in FGF-23 producing cells in bone [32]. *Fgfr1*^*DT-cKO*^, in contrast, had normal spinal and tail development, but demonstrated diminished cortical thickness, similar to *TRPV5*^*-/-*^ mice with calcium wasting and elevated PTH levels [44]. Interestingly, elevations in PTH observed in *Fgfr1*^DT-cKO^ mice did not result in secondary increments in serum FGF-23 levels. PTH effects on FGF-23, however are variable and context dependent [45]. Although we don’t have a specific explanation for why elevations in PTH did not stimulate FGF-23 in *Fgfr1*^DT-cKO^_,_ recent studies implicate a role for α-Klotho as a factor that regulates FGF-23 in osteoblasts [46]; consequently it is tempting to speculate that the reductions in α-Klotho observed in *Fgfr1*^DT-cKO^ may have contributed to the refractoriness to PTH stimulation of FGF-23.

In conclusion, the current observations suggest the following physiological schema. FGF-23 directly inhibits phosphate reabsorption in the proximal tubule through activation of FGFR1 (and to a lesser extent FGFR3 and FGFR4), and suppresses 1,25(OH)_2_D production mainly through activation of FGFR3 and FGFR4 [20]; in turn, reduced 1,25(OH)_2_D production by the kidney limits gastrointestinal calcium absorption and leads to secondary increments in PTH. Increased FGF-23 and PTH work together to inhibit proximal tubular phosphate reabsorption through FGFRs and PTH receptors in the proximal tubule. Similarly, both FGF-23 and PTH have concurrent effects to enhance renal calcium reabsorption in the distal tubule through their respective receptors. The consequent increase in the renal reabsorption of calcium offsets the hypocalcemic effects of reduced 1,25(OH)_2_D, and in doing so, allows PTH and FGF-23 to increase the renal excretion of phosphate, while maintaining calcium homeostasis. In this schema, increments in serum calcium or excess 1,25(OH)_2_D suppress PTH production by the parathyroid gland, but stimulate FGF-23 production by bone [4], creating a scenario where elevations of FGF-23 can offset the loss of PTH on urinary phosphate and calcium excretion by directly inhibiting proximal tubule phosphate, stimulating distal tubular calcium reabsorption, and reducing the renal production of 1,25(OH)_2_D.

## Supporting Information

S1 Fig Immunohistochemistry of FGFR1 expression in the kidney of WT, *Fgfr1*^PT-cKO^, and *Fgfr1*^DT-cKO^ mice. FGFR1 staining was found in both proximal and distal tubules in kidney sections derived from wild-type mice. *Fgfr1*^DT-cKO^ mice showed loss of FGFR1 staining in the distal tubules and persistent FGFR1 staining in the proximal tubule. The FGFR1 expression pattern was reciprocal in *Fgfr1*^PT-cKO^ mice, with loss of FGFR1 in the proximal tubule and continued expression in the distal tubular segments. The proximal tubules are identified by FGFR1 expression in luminal brush border membranes. The different structures are indicted as follows: proximal tubules (PT), distal tubules (DT), glomeruli (G).
(PDF)

S2 Fig Comparison of TRVP5 antibodies. A and B. Western blot analysis of expression of TRPV5 from membrane proteins prepared from kidney cortex of 16-weeks-old control, *Fgfr1*^PT-cKO^, or *Fgfr1*^DT-cKO^ mice. TRPV5 expression in membrane protein was detected by both the anti-TRPV5 antibody from Alomone (A) and from Santa Cruz (B). C and D. Immunohistochemical staining of TRPV5 expression in kidney sections of 16-weeks-old control, *Fgfr1*^PT-cKO^, or *Fgfr1*^DT-cKO^ mice. TRPV5 expression was decreased in distal tubule of *Fgfr1*^DT-cKO^ mice compared to WT control mice using either Alomone (C) or Santa Cruz TRPV5 (D) antibodies.
(PDF)

S3 Fig Npt2a protein expression in membrane-derived proteins from the kidney cortex of 16-weeks-old WT, *Fgfr1*^PT-cKO^, and *Fgfr1*^DT-cKO^ mice. Western blot analysis was performed to assess expression of Npt2a using the Abcam anti-Npt2a antibody. To validate the antibody, protein samples isolated from Npt2a overexpressing MDCK cells and from pcDNA3.1 vector-transfected MDCK cells that lack Npt2a expression [35] were used as positive and negative controls, respectively. Expression of Npt2a was decreased in *Fgfr1*^DT-cKO^ mice and increased in *Fgfr1*^PT-cKO^ mice.
(PDF)
