# Supplementary figures and images for: Conditional Deletion of Fgfr1 in the Proximal and Distal Tubule Identifies Distinct Roles in Phosphate and Calcium Transport
Source: PLoS One. 2016 Feb 3;11(2):e0147845. doi: 10.1371/journal.pone.0147845 (PMC4739706; doi:10.1371/journal.pone.0147845)

FGFR1

WT

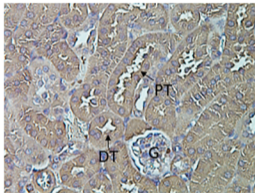

R1 PT cKO

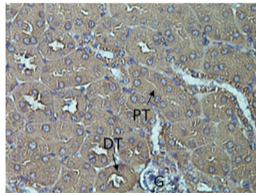

R1 DT cKO

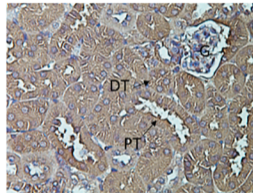

Supplement: S1 Fig — FGFR1 staining was found in both proximal and distal tubules in kidney sections derived from wild-type mice. Fgfr1DT-cKO mice showed loss of FGFR1 staining in the distal tubules and persistent FGFR1 staining in the proximal tubule. The FGFR1 expression pattern was reciprocal in Fgfr1PT-cKO mice, with loss of FGFR1 in the proximal tubule and continued expression in the distal tubular segments. The proximal tubules are identified by FGFR1 expression in luminal brush border membranes. The different structures are indicted as follows: proximal tubules (PT), distal tubules (DT), glomeruli (G). (PDF) [file pone.0147845.s001.pdf]

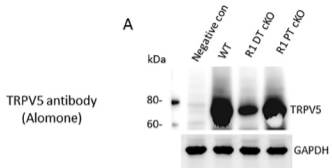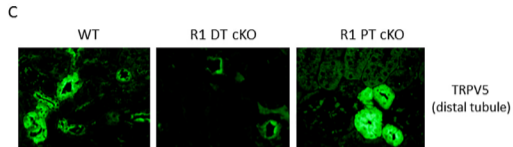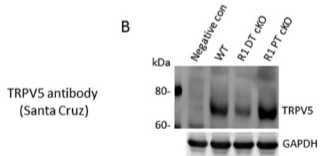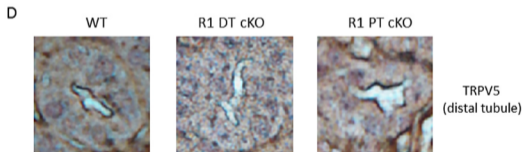

Supplement: S2 Fig — A and B. Western blot analysis of expression of TRPV5 from membrane proteins prepared from kidney cortex of 16-weeks-old control, Fgfr1PT-cKO, or Fgfr1DT-cKO mice. TRPV5 expression in membrane protein was detected by both the anti-TRPV5 antibody from Alomone (A) and from Santa Cruz (B). C and D. Immunohistochemical staining of TRPV5 expression in kidney sections of 16-weeks-old control, Fgfr1PT-cKO, or Fgfr1DT-cKO mice. TRPV5 expression was decreased in distal tubule of Fgfr1DT-cKO mice compared to WT control mice using either Alomone (C) or Santa Cruz TRPV5 (D) antibodies. (PDF) [file pone.0147845.s002.pdf]

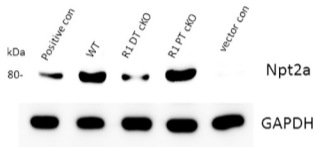

Supplement: S3 Fig — Western blot analysis was performed to assess expression of Npt2a using the Abcam anti-Npt2a antibody. To validate the antibody, protein samples isolated from Npt2a overexpressing MDCK cells and from pcDNA3.1 vector-transfected MDCK cells that lack Npt2a expression [35] were used as positive and negative controls, respectively. Expression of Npt2a was decreased in Fgfr1DT-cKO mice and increased in Fgfr1PT-cKO mice. (PDF) [file pone.0147845.s003.pdf]
